# Low grade T1 non-muscle invasive urothelial carcinoma in pediatric patient: A rare case report and management dilemma

**DOI:** 10.1016/j.eucr.2026.103406

**Published:** 2026-03-14

**Authors:** Naser El-Mefleh, Mohamad Alhardan, Hassan Al-Hussein, Abd Alrahman Haj Eisa, Osama AL-Omar

**Affiliations:** a Department of Surgery, Division of Pediatric Surgery, Aleppo University Hospital, Aleppo, Syria; b Faculty of Medicine, University of Aleppo, Aleppo, Syria; c Department of Urology, Police Hospital, Damascus, Syria; d Kidney Transplantation Unit, Al-Mouwasat Hospital, Damascus, Syria; e Urology Surgery Department, Al-Shifa Hospital, Idlib, Syria; f Department of Urology, Police Hospital, Aleppo, Syria; g Department of Urology, Division of Pediatric Urology, West Virginia University (WVU) Medicine Children, Morgantown, WV, USA

**Keywords:** Pediatric bladder cancer, Urothelial carcinoma, TURBT, Hematuria, Transurethral resection, BCG, PUNLMP

## Abstract

Papillary urothelial carcinoma (UC) is rare in the pediatric population, with its presentation and etiology differing significantly from adults, posing unique diagnostic challenges. This case report describes a 10-year-old female with abdominal pain and microscopic hematuria. Ultrasonography identified a hypoechoic lesion, and subsequent cystoscopy confirmed a 1 cm exophytic tumor. Histopathology revealed a papillary UC with focal lamina propria invasion. The postoperative course was uneventful with no evidence of recurrence. Pediatric low-grade UC is a distinct entity with an excellent prognosis following complete resection. This case underscores the need to consider malignancy in the differential diagnosis of pediatric hematuria.

## Highlights

• Diagnostic Challenge: Microscopic hematuria in a child requires complete urological evaluation to exclude rare malignancies.
• Therapeutic Conservatism: Adjuvant BCG was omitted due to the tumor's low progression risk and therapy's side effects.
• Vigilant Surveillance: A cautious approach was prioritized to enable early detection of recurrence and preserve excellent prognosis.

## 1 Introduction

Bladder cancer ranks among the most common malignancies globally, with the ninth highest incidence and the thirteenth highest mortality rate among all cancers.^1^ Urothelial carcinomas (UC) constitute the vast majority of cases, with most (75%) being non-muscle-invasive (NMIBC) at diagnosis, confined to the mucosa (Ta, carcinoma in situ) or submucosa (T1).^2^ In adults, established risk factors include smoking and occupational chemical exposures, with a long latency period.^3^ Clinical evaluation is guided by risk stratification systems, such as from the American Urological Association (AUA), which consider age, sex, smoking history, and degree of hematuria.^4^ The diagnostic and therapeutic cornerstone for NMIBC is transurethral resection of the bladder tumor (TURBT), which provides staging, grading, and initial treatment.^5^

In stark contrast, bladder carcinoma is exceedingly rare in the first two decades of life, with an incidence of 0.1%–0.4%.^6^ Although the pediatric cases may present similar to adult cases (microscopy/gross hematuria and irritative lower tract symptoms), but typically lack the risk factors seen in adults and present a different histopathological spectrum, where rhabdomyosarcoma is more common and urothelial tumors are often solitary, low-grade lesions with an indolent course.^7^, ^8^, ^9^

This report details a case of Low grade (LG) pT1 urothelial carcinoma in a 10-year-old girl who presented with abdominal pain and microscopic hematuria, highlighting the diagnostic challenges and management dilemmas in pediatric NMIBC, where adult guidelines must be carefully adapted.

## 2 Case presentation

A 10-year-old, otherwise healthy female (30 kg) presented to her pediatrician with acute, non-specific abdominal pain. Physical examination was unremarkable. Initial laboratory studies were within normal limits, but urinalysis revealed microscopic hematuria. Abdominal ultrasonography identified a hypoechoic lesion on the left lateral wall of the bladder. The patient was referred for urological evaluation. Under general anesthesia, diagnostic cystoscopy was performed with a 12Fr cystoscope, revealing a 1 cm exophytic lesion lateral to the left ureteral orifice (Video 1). A complete transurethral resection of the bladder tumor (TURBT) was performed using a 13Fr resectoscope, including the detrusor muscle. The procedure was uneventful; the urinary catheter was removed 2 h postoperatively, and the patient was discharged on the second postoperative day.

Supplementary data related to this article can be found online at https://doi.org/10.1016/j.eucr.2026.103406

The following are the Supplementary data related to this article:

Multimedia component 1 Video 1: Cystoscopy demonstrates a 1 cm exophytic lesion on the left lateral bladder wall, near the ureteric orifice. Multimedia component 1

**Fig. 1:**
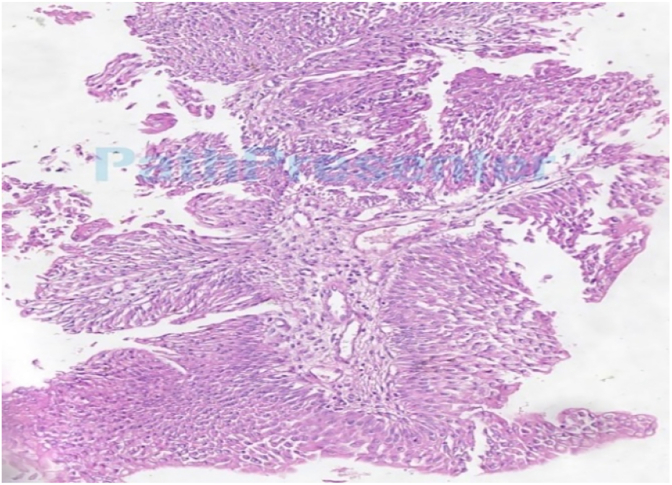
Histopathological examination (H&E stain, 100x) revealed a papillary urothelial neoplasm with fibrovascular cores lined by urothelium with minimal cytological atypia, consistent with PUNLMP. Focal invasion into the lamina propria was identified, warranting a final pathological stage of pT1. There is minimal detrusor muscular component with no invasion.

A multidisciplinary tumor board discussion was held. Given the patient's age, the low-grade/PUNLMP histology (Fig. 1), and the known favorable biology of pediatric urothelial tumors, the decision was made to forgo adjuvant intravesical Bacillus Calmette-Guérin (BCG) therapy. A surveillance protocol was instituted. A second-look TURBT performed 5 weeks after the initial resection showed no residual tumor. Surveillance included cystoscopies at 1.5, 6, and 16 months post-resection, all of which were negative for recurrence. Serial renal and bladder ultrasounds have also shown no evidence of disease.

## 3 Discussion

Pediatric bladder malignancies are extremely rare with most cases occurring sporadically without identifiable risk factors.^7^ The scarcity of cases means there is a lack of high-level evidence from pediatric-specific clinical trials to guide management.^7^ Therefore, current clinical practice relies on risk-adapted, multimodal therapy protocols established by expert collaborative groups 7. For the very rare urothelial tumors, clinicians may reference adult guidelines, though these are not validated for the pediatric population.^10^

This case illustrates the unique considerations in diagnosing and managing NMIBC in a pediatric patient. The initial presentation of microscopic hematuria and abdominal pain was non-specific, underscoring the need for a high index of suspicion to initiate appropriate imaging and endoscopic evaluation.^6^

### 3.1 Pathological classification and prognostic implications

During the last 40 yr, different bladder tumor-grading systems have been introduced, with generally good acceptance. The evolution of grading systems from the WHO 1973 (G1-G3) to the contemporary WHO 2004/2016/2022 classifications reflect an effort to improve diagnostic reproducibility and prognostic relevance.^11^^,^^12^ Using a histoanatomical approach, the pT1 substage is evaluated according to tumor invasion within or beyond the muscularis mucosae or the large vessels of the lamina propria. The micrometric approach is based on the actual measurement of the invasive component.^12^ The tumor was best classified as a PUNLMP with focal lamina propria invasion (pT1). According to the WHO 2016 system, pTa and pT1 tumors are graded into LG and HG and all detrusor muscle-invasive urothelial carcinomas are considered to be HG tumors.^5^^,^^13^ The PUNLMP category, retained from the WHO 2016 system and reaffirmed in the 2022 update, is particularly relevant in younger patients as it does not carry the label “carcinoma,” yet it is not a completely benign lesion and can recur.^12^^,^^13^ While molecular differences exist, the distinction from low-grade (LG) non-invasive papillary carcinoma (pTa LG) is morphological.^10^^,^^13^, ^14^, ^15^ In children, even pT1 disease appears to have a markedly more indolent course than in adults.^16^ Due to the lack of a pediatric-specific staging system for urothelial carcinoma, the same staging and grading systems used in adult urology are applied to pediatric cases**.**

### 3.2 Management dilemma: adult guidelines vs. pediatric reality

Our case of low grade T1 non-muscle invasive bladder cancer (LGT1 NMIBC) is considered an intermediate risk for recurrence and progression into muscle invasive cancer according to the AUA guidelines. In adults, the AUA recommendation for intermediate risk NMUC like our case includes a one dose of intravesical chemotherapy in the first 24 hours, followed by a second TURBT and strong consideration of adjuvant BCG therapy.^16^, ^17^, ^18^ Currently, there is no pediatric guidelines for urothelial cancer for obvious reasons. However, pediatric literature consistently reports an excellent prognosis for low-grade tumors with complete resection alone, making post-resection surveillance is more appropriate for pediatric patients.^16^^,^^17^ The risk of progression and recurrence is minimal, while the potential side effects of BCG (including systemic complications in <5% of adults) are a significant concern in a developing child.^11^ Therefore, we, like others, opted for a conservative approach, forgoing BCG.

### 3.3 Surveillance strategy and practical challenges

Surveillance for pediatric NMIBC is not well-defined. We adapted an adult low-risk protocol (initial cystoscopy at 3 months) but intensified early follow-up due to the pT1 stage.^17^^,^^19^ Our schedule (cystoscopy at 1.5, 6, and 16 months) aimed to balance vigilance with practicality. This case also highlights a real-world challenge: strict adherence to surveillance can be hindered by logistical obstacles. In our setting, socio-political factors impacted scheduling, yet the absence of obvious risk factors for bladder cancer and lack of recurrence over 16 months reinforces the tumor's indolent nature and validates the conservative strategy.

## 4 Conclusion

Pediatric papillary urothelial carcinoma, even at a pT1 stage, is a distinct clinical entity characterized by low-grade histology and an excellent prognosis following complete TURBT. Management should be individualized, balancing oncologic principles derived from adult guidelines against the unique biology of the pediatric disease and the long-term risks of therapy. A second TURBT is prudent for staging accuracy, but adjuvant BCG can often be safely omitted in favor of a structured surveillance protocol. This case adds to the reported sporadic cases of urothelial carcinoma in pediatric patients with the hope for possible future standardization of their management and post-operative management and reinforces the critical importance of including malignancy in the differential diagnosis of pediatric hematuria to ensure timely diagnosis and appropriate' individualized management.

## CRediT authorship contribution statement

**Naser El-Mefleh:** Writing – review & editing, Writing – original draft, Visualization, Validation, Supervision, Resources, Project administration, Methodology, Formal analysis, Data curation, Conceptualization. **Mohamad Alhardan:** Writing – review & editing, Writing – original draft, Validation, Supervision, Resources, Project administration, Investigation, Formal analysis, Data curation, Conceptualization. **Hassan Al-Hussein:** Writing – review & editing, Writing – original draft, Validation, Resources, Methodology, Investigation, Formal analysis, Data curation, Conceptualization. **Abd Alrahman Haj Eisa:** Writing – review & editing, Writing – original draft, Formal analysis. **Osama AL-Omar:** Writing – review & editing, Writing – original draft.

## Ethical approval

Ethical approval for this case report was waived by Institutional Review Board.

## Patient consent

Written informed consent was obtained from the patient's legal guardian(s) for publication of this case report and any accompanying images/videos. A copy of the written consent is available for review by the Editor-in-Chief of this journal.

## Funding source

This research did not receive any specific grant from funding agencies in the public, commercial, or not-for-profit sectors.

## Conflict of interest

All authors declare that they have no conflicts of interest.
